# Genetic heterogeneity of L-Zagreb mumps virus vaccine strain

**DOI:** 10.1186/1743-422X-5-79

**Published:** 2008-07-10

**Authors:** Tanja Kosutic-Gulija, Dubravko Forcic, Maja Šantak, Ana Ramljak, Sanja Mateljak-Lukacevic, Renata Mazuran

**Affiliations:** 1 Department for Research and Development, Institute of Immunology Inc, Rockefeller Street 10, Zagreb, Croatia; 2 Department of Viral Vaccines, Institute of Immunology Inc, Rockefeller Street 10, Zagreb, Croatia; 3 Quality Control Department, Institute of Immunology Inc, Rockefeller Street 10, Zagreb, Croatia

## Abstract

**Background:**

The most often used mumps vaccine strains Jeryl Lynn (JL), RIT4385, Urabe-AM9, L-Zagreb and L-3 differ in immunogenicity and reactogenicity. Previous analyses showed that JL, Urabe-AM9 and L-3 are genetically heterogeneous.

**Results:**

We identified the heterogeneity of L-Zagreb throughout the entire genome. Two major variants were defined: variant A being identical to the consensus sequence of viral seeds and vaccine(s) and variant B which differs from variant A in three nucleotide positions. The difference between viral variants in L-Zagreb strain is insufficient for distinct viral strains to be defined. We demonstrated that proportion of variants in L-Zagreb viral population depends on cell substrate used for viral replication in vitro and in vivo.

**Conclusion:**

L-Zagreb strain should be considered as a single strain composed of at least two variant viral genomes.

## Background

Mumps virus (MuV) genome consists of a 15 384 nt long non-segmented single-stranded negative sense RNA. The genomic RNA contains seven genes which encode nine open reading frames: NP (nucleoprotein), P (phosphoprotein, V protein, I protein), M (matrix protein), F (fusion protein), SH (small hydrophobic protein), HN (haemagglutinin-neuraminidase) and L (large protein) [1,2].

Mumps virus causes an acute systemic infection involving glandular, lymphoid and nervous tissues. Prior to the introduction of live attenuated virus vaccines, mumps virus was a leading cause of the virus-induced CNS disease [1].

Live attenuated mumps vaccines have been used worldwide since late 1960s [3,4]. Nowadays, the most often used vaccine strains are Jeryl Lynn (JL), RIT 4385, Urabe-AM9, L-Zagreb and Leningrad-3 (L-3) [5,6].

Although at the time of their development the knowledge of molecular content of mumps vaccines was not the issue, recently it has become obvious that the molecular consistency of vaccine production is not a trivial matter. Sauder et al [7] showed that the change in genetic heterogeneity at the specific genome sites can have a profound effect on neurovirulent phenotype of Urabe-AM9 strain. The RNA viral population consists of virus particles that differ from the consensus sequence in one or more nucleotides (quasisipecies), the feature that arises because of the high mutation rate of RNA-dependent RNA polymerase (RdRp) (10^-3 ^to 10^-5 ^errors per nucleotide site and replication cycle) [8,9]. Given that all mumps vaccines are quasiespecies populations, an adequate description of the vaccine virus genome should include not only the consensus sequence, but also the quantitative assessment of the existing viral variants.

Previous analyses confirmed that mumps vaccine strains JL, Urabe-AM9 and L-3 are genetically heterogeneous. JL is composed of a mixture of two distinct viral strains (JL5 and JL2) [10,11] while Urabe-AM9 represents a quasispecies mix [12,13].

L-3 vaccine strain was prepared from five mumps virus isolates combined into a single strain in 1953 [4]. It was characterized as heterogenic on the basis of plaque morphology [14] and a sequence autoradiogram with several ambiguities in P and F genes [15] but precise vaccine composition of L-3 was never published.

L-Zagreb vaccine strain was developed by further subcultivation of L-3 mumps vaccine strain in primary culture of chicken embryo fibroblast (CEF) [16]. Genetic stability at the level of the consensus sequence of the L-Zagreb vaccine strain in the course of the production process was demonstrated [17].

Here, we analyzed the detailed genetic composition of L-Zagreb vaccine strain. Due to mixture of mumps virus isolates in L-3 production and its heterogeneity we wonder about the composition of L-Zagreb strain.

By two independent cloning experiments we showed that L-Zagreb vaccine strain contains only one viral strain. However, numerous nucleotide positions showed to be heterogenic and indicating a quasispecies nature of this strain. We successfully isolated two types of viral clones: identical to consensus sequence, named as variant A, and with the nucleotide sequences different from the consensus sequence (quasispecies). The most abundant quasispecies, named variant B, was detected in all analyzed L-Zagreb samples.

Finally, we demonstrated that the heterogenic composition of L-Zagreb strain strongly depends on the number of passages and the type of the cell culture that the virus is replicating on.

## Results and discussion

### Heterogenic nucleotide positions in the L-Zagreb vaccine strain genome

The strategy for defining heterogenic positions in the L-Zagreb vaccine strain genome involved cloning of eleven overlapping PCR fragments into pUC19 plasmid vector and sequencing of resulting plasmid clones. For each fragment, two independent cloning experiments were performed in order to avoid misinterpretation of artificial heterogeneity arisen from the error of Pfu DNA polymerase used for fragment amplification [18]. Twenty and ten clones were analyzed in the first and the second experiment, respectively. Cloned genome fragments were compared to the consensus sequence of the L-Zagreb strain [GenBank: AY685920] in order to select clones with changed nucleotides.

**Figure 1:**
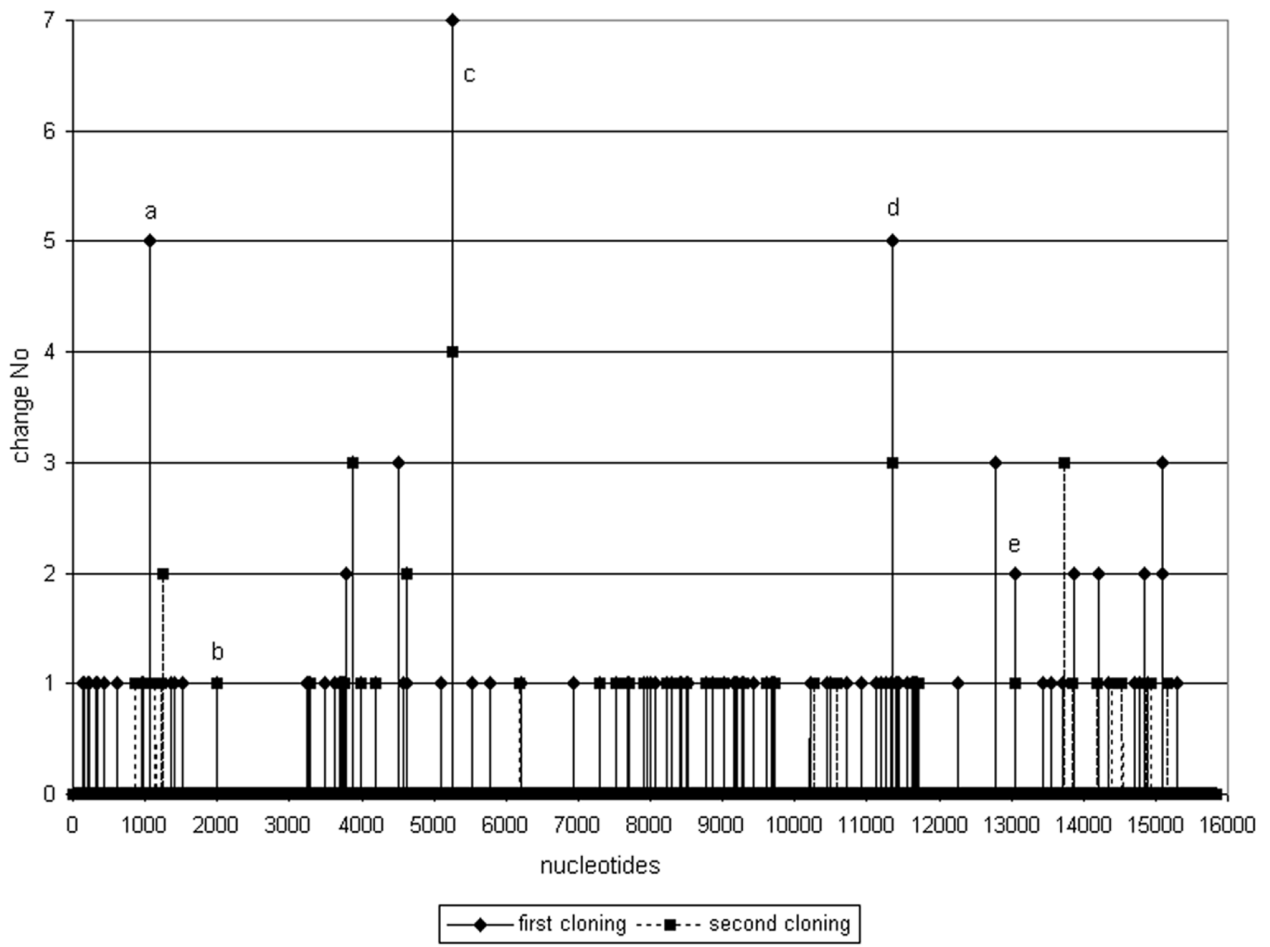
Schematic presentation of mumps virus genome with changed nucleotides. Black triangles represents changes detected in first experiment while gray squares represents changes detected in second experiment. Six nucleotides positions are detected as changed in both experiments: a = nt 1059, b = nt 1073, c = nt 1996, d = nt 5261, e = nt 11345, f = nt 13054.

As a result, 88 and 49 nucleotides different from the consensus sequence were identified throughout a complete genome of L-Zagreb strain, in the first and the second experiment respectively (Fig 1). The distribution of the heterogenic positions seem to be at random except for the region between approx. 2000 and 3000 nt which corresponds to almost a complete coding region of P gene (which spans region 1979–3152 nt) where no heterogeneity was found (Fig 1).

By comparing changed nucleotides of the first and the second cloning experiment, alterations of six nucleotides (1059, 1073, 1996, 5261, 11345 and 13054) were identified in both cloning experiments in one or more clones. Alterations G1059A was identified in 1/20 and 1/10 sequenced plasmid clones, G1073T in 5/20 and 1/10; A1996C in 1/20 and 1/10, C5261T in 7/20 and 4/10, G11345T in 5/20 and 3/10 plasmid clones and alteration C13054A was identified in 2/20 and 1/10 plasmid clones (Fig 1).

Other 125 changed nucleotides were found within a single clone in one of the experiments (Fig 1). They could easily be considered as possible heterogeneities in L-Zagreb strain, although it should not be ruled out that some of them originated from a low error rate of the enzymes involved in fragment amplification.

### Heterogeneity of isolated viral clones

Based on the above results it may be predicted that there are different viral variants constituting L-Zagreb strain. However, solely by defining heterogenic positions in cloned fragments it was not possible to identify the variants. Therefore, viral clones of the L-Zagreb strain were plaque isolated in the Vero cell culture and additionally subcultivated once in the same cell culture. Twelve viral clones were identified by sequencing in regions which included six heterogenic positions identified in cloning experiments: 1059, 1073, 1996, 5261, 11345 and 13054. The analysis of the picked viral clones indicated two viral variants, herein named variant A and variant B. Variant A was shown to be identical to the consensus sequence in all 6 positions: 1059G, 1073G, 1996A, 5261C, 11345G, and 13054C. Variant B differed from the consensus sequence in positions 1073T, 5261T and 11345T.

Seven out of 12 analyzed viral clones represented variant A and five out of 12 represented variant B.

In the process of determination of the viral variants we used only 6 positions identified as heterogenic by the cloning experiments. As that could limit the criteria for defining the variants, a complete genome of one viral clone belonging to variant B was sequenced and compared to the consensus sequence. Again, the differences were only in positions 1073, 5261 and 11345, what confirmed our criteria for defining variant B as adequate. Also it confirmed the presence of Ts at positions 1073, 5261 and 11345 as the genetic marker of variant B versus variant A.

Three positions (1073, 5261 and 11345) which differentiate variants A and B caused diversity in amino acid composition of the N (310 aa, A→S), F (239 aa, T→I) and L (970 aa, V→L) protein. Although these amino acids are not part of any known functional domains it is difficult to predict biological impact of these differences since the molecular structures of mumps virus proteins are not well characterized.

Heterogenic positions 1059, 1996 and 13054 were not detected in any of the twelve analyzed viral clones. Since they were identified in both cloning experiments, they can not be considered as misinterpretation due to the erroneous nucleotide incorporation in the amplification process. They should rather be considered as differential positions of viral variants which coexist with variant A and B, but at low amount. Although heterogenic positions represented by low amount seem at random and not relevant for the virus, the *in vivo *impact of these minor variants should not be minimized. Previous study [7] indicated the existence of multiple genetic markers and a need for evaluation of a total viral population instead of putting the relevance of a consensus sequence in foreground.

Since L-Zagreb vaccine strain originated from L-3 vaccine strain [16], we compared the nucleotide sequences of both variants to the consensus sequence of L-3 [GenBank: AY508995] (Table 1). Comparison of the entire genome of L-3 strain with the variant A and variant B genomes showed difference in five and eight nucleotides, respectively (Table 1). Since the heterogeneity of L-3 strain is not completely resolved it is not possible to define when the heterogenic positions in L-Zagreb strain occurred: were they newly created by mutational process in the course of adaptation in primary CEF culture or was it the selection of preexisting minor quasispecies of L-3 strain which occurred when Japanese quail embryo fibroblast culture was replaced by primary CEF.

**Table 1:** Nucleotide and amino acid differences between L-3 vaccine strain and variant A (var A) and variant B (var B) of L-Zagreb strain.

Gene	nt position	aa position	L3:varA	L3:varB
*leader*	14 a→t 30 a→g	--- ---	≠ ≠	≠ ≠
NP	1059 g→a 1073 g→t	305 310	≠ =	≠ ≠
F	5037 a→g 5261 c→t	NC 239	≠ =	≠ ≠
L	11345 g→t 15325 t→a	970 NCR	= ≠	≠ ≠

(aa amino acid, NC not changed, NCR non-coding region, ≠ non-identical, = identical)

High level of difference between JL5 and JL2 (414 nt, 87 aa), demonstrated JL vaccine as a mixture of two distinct viral strain [11]. In contrast to JL, the two most abundant viral variants in L-Zagreb strain differ in three nucleotide positions (three aa) what is an insufficient variation for two distinct viral strains to be defined. L-Zagreb strain should be considered as a single strain composed of a number of quasispecies viral genomes. Thus, L-Zagreb strain is similar to Urabe-AM9 vaccine strain which consists of at least two viral variants with minor genetic changes [19,12,13].

### Screening of heterogeneity in the L-Zagreb seeds and vaccine lots

Here, the same sample was used for both cloning experiments and plaque isolation. Due to the high genetic plasticity of RNA viruses one could easily assume that the identified heterogenic positions reflect only the composition of that sample and is not the intrinsic feature of L-Zagreb vaccine strain.

Therefore the heterogeneity of L-Zagreb strain was analyzed in vaccine seeds (master and working) and two final vaccine batches. The T in heterogenic position 5261 in the F gene is located within the SspI restriction site while the C in the same position eliminates this restriction site. The existence or the absence of restriction site facilitated the use of PCR-RFLP assay as an adequate method for distinguishing the two variants, A and B. A 321 bp uncleaved fragment indicated variant A while a 219 bp cleaved fragment indicated variant B.

Heterogenic positions 1073 and 11345 which are also genetic markers of variant B were unsuitable for RFLP assay. However, it was proved above that these three positions represent the marker of variant B.

**Figure 2:**
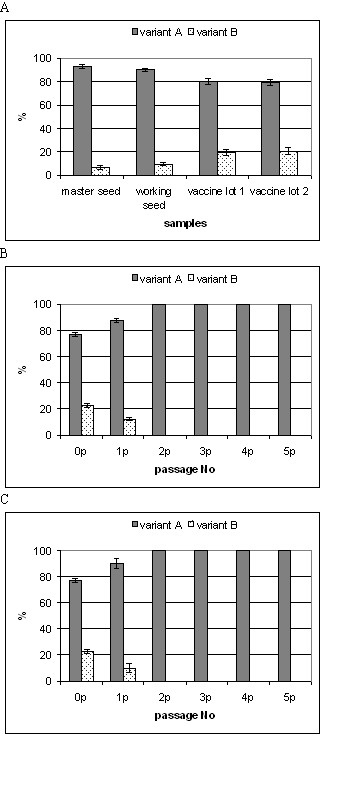
The proportion of variant A and variant B detected in position 5261 in mumps virus genome. The L-Zagreb samples were propagated on (a) CEF, (b) Vero, and (c) SH-SY5Y cell cultures. Data from two experiments are presented.

Both variants were detected in all four viral samples, but at different proportions (Table 2, Fig 2A). The master seed consisted of 93.0 ± 1.8% variant A and 7.0 ± 1.8% variant B. In the working seed the proportion of variant B increased to 9.7 ± 1.1% and the proportion of variant A decreased to 90.3 ± 1.1%. The two final vaccine batches contained even lower proportions of variant A (80.4 ± 2.6% and 79.4 ± 2.9%, respectively) and higher proportions of variant B (19.6 ± 2.6% and 20.6 ± 2.9%, respectively) in comparison to the viral seeds.

**Table 2:** The proportion of variants A and B in the L-Zagreb samples: L-Zagreb master seed, L-Zagreb working seed and L-Zagreb final vaccine lots 1 and 2.

L-Zagreb sample	Variant (%)*	
	
	A	B
Master seed	93.0 ± 1.8	7.0 ± 1.8
Working seed	90.3 ± 1.1	9.7 ± 1.1
Final vaccine lot 1	80.4 ± 2.6	19.6 ± 2.6
Final vaccine lot 2	79.4 ± 2.9	20.6 ± 2.9

* the arithmetic mean ± SD of four independent PCR-RFLP experiments

Altogether, these data indicate that different L-Zagreb vaccine strain samples are heterogenic in the same nucleotide positions. Although the stability of the L-Zagreb consensus sequence of the master seed and a final vaccine batch was confirmed [17], it is clear that the quasispecies content is changing by the passage number.

### Influence of different cell culture on heterogeneity of L-Zagreb

Different production stages of L-Zagreb vaccine analyzed above originated through increasing number of passages in primary chicken embryo fibroblasts (CEF). Analysis of the proportion of variants A and B in the vaccine samples clearly shows that CEF somewhat favor replication of variant B over variant A (Fig 2A). The influence of selected cell line on replication efficiency of viral clones and thus a genetic heterogeneity of the entire viral sample was also reported previously [20].

A limited number of serial passages of L-Zagreb vaccine strain in Vero and SH-SY5Y cell line further confirmed the influence of the cell culture selection on the genetic composition of L-Zagreb strain (Fig 2B and 2C). Cell supernatants from each of five passages were analyzed by PCR-RFLP assay in position 5261. The original sample (p0) contained 77.7 ± 1.5% of variant A and 22.3 ± 1.5% of variant B. The first passage decreased the proportion of variant B in both Vero and SH-SY5Y cells to 12.2 ± 1.5% and 10.0 ± 3.6%, respectively (Fig 2B and 2C, respectively). Surprisingly, the proportion of variant B was diminished to an undetectable level already in the following passage and remained undetectable for the next three passages in both cell types (Fig 2B and 2C). This clearly shows that both Vero and SH-SY5Y cells, in contrast to CEF, promote the replication of variant A leading to the loss of variant B, the second most abundant genomic variant in L-Zagreb strain.

However, when propagated as a genetically homogenous viral clone, variant B was able to replicate in both cell types up to the same extent as variant A (data not shown) indicating that variant A either uses up the cell capacity to produce viral particles faster then variant B or it actively suppresses the replication of variant B by an unknown mechanism.

## Conclusion

Our data confirm heterogeneous nature of the L-Zagreb mumps vaccine strain and permanent existence of minor variant B whose replication is favored by the production cell culture. Variant B differed from consensus sequence in only tree nucleotides what give us reason to conclude that vaccine strain L-Zagreb is composed of only one mumps viral strain. Also we detected low represented variant changed in nt positions 1059, 1996 and/or 13054.

Sauder et al. [7] showed that changes in the neurovirulent phenotype were merely associated with the changes of the level of genetic heterogeneity. In addition to cell substrate, serial passaging of mumps virus could reduce or increase neurovirulent phenotype of the virus.

Due to its heterogenic profile, examined L-Zagreb vaccine lot could serve as comparator in investigations of genetic profile of L-Zagreb postvaccinal mumps virus [21] or L-Zagreb horizontally transmitted mumps virus [22].

## Materials and methods

### Viral material and cell culture

L-Zagreb master and working seeds were stored at -80°C until used for RNA extraction. Freeze-dried L-Zagreb vaccine lots 1 and 2 were reconstituted in 250 μl of water prior to RNA extraction or in 500 μl of sterile water prior to plaque assay. All viral materials were produced at the Institute of Immunology Inc, Zagreb, Croatia.

Vero cell culture (African green monkey kidney cells) was obtained from the American Type Culture Collection (USA) and cultured in minimal essential medium (MEM-H) (AppliChem, Germany) supplemented with 10% fetal calf serum (FCS) (Moregate, Australia) and neomycin 50 μl/ml (Gibco-BRL, USA). Human neuroblastoma cell culture, SH-SY5Y was obtained from European Collection of Cell Cultures, (UK) and cultured in D-MEM medium supplemented with 10% FCS and neomycin 50 μl/ml.

### RT-PCR

Viral RNA was extracted from 250 μl of viral seeds or reconstituted vaccine as reported by Chomczynski and Mackey [23].

cDNA synthesis and amplification of the complete genome segmented in eleven overlapping fragments, were performed as described in Ivancic et al [17]. Briefly, RNA was reverse transcribed with random hexamers and MuLV (Applied Biosystems, USA) for 1 h at 42°C. Amplification was performed with the whole reverse transcription mix containing 2.4 U Pfu DNA polymerase (Promega, USA) in a total volume of 100 μl.

### Molecular cloning

Successful amplification of fragments of the expected size was confirmed by electrophoresis in 1% agarose gels. The fragments were purified using a Qiaquick kit (Qiagen, Germany) and cloned into pUC19 vector (New England Biolabs, USA) using protocol for blunt-end cloning described in pMOSBlue Blunt Ended Cloning Kit (GE Healthcare, UK) with modification.

Briefly, plasmid DNA was linearised with *SmaI *and dephosphorylated using alkaline phosphatase (Roche, Germany). DNA fragments were converted into blunt, phosphorylated products in a one step reaction using PK enzyme mix from pMOSBlue Blunt Ended Cloning Kit (GE Healthcare, UK). Followed by brief heat incubation the product was ligated overnight at 16°C into dephosphorylated blunt ended pUC19.

*Escherichia coli *strain DH5amcrAB (Life Technologies Ltd, USA) was transformed with the ligation mix. Transformed bacteria were picked out using blue-white selection. Plasmid DNA was isolated from overnight cultures by alkaline lysis [24].

### DNA sequencing

Segments of mumps virus genome were sequenced either upon cloning into pUC19 vector or directly as PCR products of isolated viral clones by using M13 FSP (5'gctggcgaaagggggatgtg3') and M13 RSP (5'cactttatgcttccggctcg3') primers or mumps virus specific primers [17]. Sequencing was performed on a 3130 Genetic Analyzer (Applied Biosystems, USA) using the BigDye Terminator v3.1 Cycle Sequencing Kit (Applied Biosystems, USA) according to the protocol recommended by the manufacturer. Obtained sequences were analyzed by CloneManager Suite software (Scientific&Educational Software, USA).

### Isolation of viral clones

The 8 × 10^5 ^Vero cells were grown in six-well plates for 24 h. Virus was diluted in MEM-H with 2% FCS and neomycin. Aspirated cell monolayer was infected with 0.5 ml of viral suspension. After 1 h at 37°C viral suspension was aspirated, cells were washed twice with PBS and 3 ml of overlay I (1 v/v 2 × MEM-H with 10% FBS without phenol red and 1 v/v 1.4% Noble agar (Sigma, USA)). Plates were incubated at 37°C in a humidified atmosphere of 5% CO_2_. After five days 1 ml of overlay II (0.02% Neutral red (Sigma, USA) plus overlay I) was added. Plates with solidified overlay II were incubated at 37°C for the next 24 h in a humidified atmosphere of 5% CO_2_. Viral clones were cut out and transferred on Vero cells for one additional passage in order to prepare viral suspension for the viral identification by DNA sequencing.

### Virus passages in cell cultures

Vero and SH-SY5Y were grown in six-well plates for 24 h. Cells were infected with monovalent L-Zagreb vaccine at m.o.i. 0.05 and incubated at 37°C for 1 h. Cells were then washed twice with PBS. Infected Vero and SH-SY5Y cells were incubated in MEM-H or D-MEM, respectively, with 2% FCS and neomycin at 37°C in a humidified atmosphere of 5% CO_2_. After 48 h the medium was collected and used for further passage and viral identification by DNA sequencing. Each consecutive passage was done with 1/10 of collected culture medium. Five consecutive passages in both cell lines were performed.

### PCR-RFLP assay

PCR products were purified by using QIAquick kit (Qiagen, Germany) and cleaved in a reaction mixtures consisting of 0.05 μg of PCR product, 10 U of *SspI *(GE Healthcare, UK) and 1× cleavage buffer in a total volume of 25 μl. The reaction was carried out overnight at 37°C. Cleaved PCR product was diluted 1:10 with water, and 1 μl was mixed with 0.5 μl of GS-500 LIZ size standard (Applied Biosystems, USA) and 9.5 μl of HiDi formamide (Applied Biosystems, USA). The mixture was denaturated at 95°C for 2 min followed by cooling on ice. Electrophoresis of cleaved PCR products was performed on 3130 Genetic Analyzer (Applied Biosystems, USA) using POP7 polymer (Applied Biosystems, USA). Analyses of PCR products were done by GeneMapper (Applied Biosystems, USA) software using peak area data [25].

## Competing interests

The authors declare that they have no competing interests.

## Authors' contributions

TKG participated in the conception of the study, performed the majority of the experiments and wrote the manuscript. DF helped in the conception of the study, its design and coordination and helped to draft the manuscript. MS participated in the molecular cloning, the nucleic acid sequencing and sequence alignment and helped to draft the manuscript. SML and AR prepared all mumps virus samples. RM participated in study design and helped to draft the manuscript. All authors read and approved the final manuscript.
